# A randomised controlled feasibility trial of guided parent-delivered cognitive behaviour therapy for children’s perfectionism

**DOI:** 10.1007/s00787-026-03131-2

**Published:** 2026-07-13

**Authors:** Tracey D. Wade, Sarah J. Egan, Maya Jabs, Madelaine de Valle, Roz Shafran

**Affiliations:** 1 Flinders University Institute of Mental Health and Wellbeing, Flinders University, Adelaide, Australia; 2 Enable Institute, Faculty of Health Sciences, Curtin University, Perth, Australia; 3 Discipline of Psychology, School of Population Health, Curtin University, Perth, Australia; 4 Great Ormond Street Institute of Child Health, University College London, London, England

**Keywords:** Perfectionism, Child, Parent, Cognitive behaviour therapy, Academic self-efficacy, Depression

## Abstract

We examined the feasibility, acceptability and preliminary efficacy of Cognitive Behaviour Therapy for Perfectionism (CBT-P) for children delivered as an online 6-session guided parent-delivered intervention (GP-CBT-P). We conducted a two-arm randomised controlled trial comparing GP-CBT-P to waitlist for parents whose child was aged 7–12 and had elevated perfectionism. Primary outcomes were feasibility and acceptability; secondary outcomes were self-reported parental and child perfectionism, school satisfaction, happiness, stress, depressive symptoms, self-efficacy, and weight/shape concern. Assessment occurred at baseline (T1), 9- (T2, end of the intervention) and 18-weeks (T3) post-randomization. There were 89 enquiries over 14 months; 49 (54%) converted to randomisation (25 in GP-CBT-P, 24 in waitlist), and 60% of parents in the intervention group completed all 6 sessions. There were no differences between groups at baseline. Intent-to-treat analysis showed parents in GP-CBT-P had significantly lower levels of perfectionism at T2 and T3 than control (respective between groups effect sizes [ES] and 95% confidence intervals [CI] of 0.95 [0.37 to 1.53] and 0.65 [0.08 to 1.21]). Compared to control, children receiving GP-CBT-P reported significantly higher school satisfaction and academic self-efficacy, and lower stress, depression and weight/shape concerns; T3 ES ranged from 0.58 to 0.82. There were no significant differences between groups on child perfectionism. GP-CBT-P could benefit from strategies to enhance feasibility and acceptability given it promoted helpful change across parent and child self-reported outcomes. Reducing parental perfectionism may be a key driver of beneficial change in children. Further investigation with larger samples and longer-term follow-up is recommended.

Perfectionism is defined as unremitting efforts to attain excessively high self-imposed standards and measuring self-worth largely in terms of accomplishment [1]. Some factor analytical evidence suggests perfectionism measures can be grouped under two higher-order dimensions [2–4]. The first is Perfectionistic Strivings, self-oriented excessively high goals. The second is Perfectionistic Concerns, the impact of mistakes, and feelings of discrepancy between one’s expectations and perceived performance.

Both types of perfectionism have been associated with psychological maladjustment in youth (children through to emerging adults), including depression, anxiety, and eating disorders [5, 6]. In children and adolescents, perfectionism has been associated with decreased social functioning and increased feelings of isolation, fear and sadness [7], and impairment in approaching valued academic goals [8, 9]. Norwegian research suggests about four of ten adolescents in mid-socioeconomic schools meet criteria for debilitating perfectionism compared to two of ten in the elite private schools [4]. Both types of perfectionism have increased linearly in college students over the last 27 years [10].

Perfectionistic Strivings is differentiated from pursuing high standards and excellence. The latter is not intrinsically unhelpful, based on evidence suggesting its association with helpful goal progress, academic behaviours and outcomes [8]. It is the *way* these high standards are approached and the *reaction* to failing to meet those standards that becomes unhelpful. When self-evaluation is overly dependent on striving and achievement, reactions include experiencing intense negative feelings when high expectations go unmet, feeling more afraid to fail than most people, taking constructive criticism defensively, being self-critical, and worrying so much about doing something imperfectly that they become immobilized.

Cognitive behavioural treatment for perfectionism (CBT-P) [11] seeks to teach people to approach achievement in ways that do not damage them and to broaden their scheme for self-evaluation. Several meta-analyses have confirmed the efficacy of CBT-P [12–15]. A large effect size within-group decrease, and moderate between-group differences, are observed across studies. Outcomes show moderate between-group effect size differences for disordered eating, anxiety and depression compared to wait-list conditions. Academic outcomes are not typically assessed despite perfectionistic concerns being maladaptive for successful learning [8].

Importantly, these interventions are scalable. They have been delivered by expert facilitators in classroom settings with children aged 10–14 years and compared to control conditions [16]. With children a significant reduction in perfectionism was observed, with a significant improvement in wellbeing at 3-month follow-up compared to controls [17]. In adolescents, the intervention produces significantly lower perfectionism, self-criticism and depression/anxiety than controls at 6-month follow-up [18]. When delivered in classrooms by teachers to young adolescents, the intervention protected against increases in anxiety, deteriorations in wellbeing (in girls), and lowered perfectionistic strivings in students with high levels of perfectionistic concerns, compared to the control condition [19]. Internet delivery of the intervention is equally as effective as face-to-face delivery [13], and results in significant and moderate reductions in perfectionism, depression and anxiety in youth aged 14–19 years [20].

A clear gap in the literature is evaluation of CBT-P with children under the age of 12 years, and the reliance on classroom delivered interventions for children aged under 12, given the challenge these concepts may pose for such a young cohort. Utilising parents to deliver the intervention increases the possibilities to support children with high levels of perfectionism and is also indicated in terms of research showing significant associations between parental and child perfectionism as well as between child perfectionism and the perception of an authoritarian parenting style [21]. While an initial feasibility and acceptability study of parent-supported CBT-P in adolescents with eating disorders has been conducted [22], no studies have examined parent-supported CBT-P in children.

Importantly, there is empirical support for this mode of delivery across several studies of childhood anxiety. Children aged 5 to 12 referred with anxiety difficulties to four National Health Service primary child and mental health services showed significant improvements in anxiety with either an online brief guided parent-delivered CBT or solution-focused brief therapy [23]. The former intervention, however, was assessed as more cost-effective. Relative to waitlist, use of written materials for parents with no therapist contact resulted in around 15% more children aged 6–12 being free of an anxiety disorder diagnosis after 12 and 24 weeks [24]. Similarly, a face-to-face group guided parent-delivered CBT for anxious children aged 7 to 14 years was found to be significantly more effective at reducing anxiety than a wait-list control group 6 weeks post-randomisation [25]. A between group effect size of 0.60 was obtained favouring the intervention group, leading to the conclusion that a brief, parent-only intervention offered a cost-effective, low intensity alternative to traditional child-focused interventions.

Given there is no evaluation of guided parent-delivered CBT-P (GP-CBT-P) to children, we chose as our primary outcomes feasibility (rate of recruitment) and acceptability (completion of the intervention). Secondary outcomes were comparative change over time in self-reported parental perfectionism and child perfectionism, school satisfaction, happiness, stress, depressive symptoms, weight/shape concern and self-efficacy. Assessment occurred at baseline (T1), 9- and 18-weeks post-randomization (T2, the end of the intervention and T3, follow-up). This represents the first occasion that academic outcomes will be examined in a randomised controlled trial design of perfectionism treatment.

## Method

### Study design

The design is a parallel-arm randomized controlled trial (RCT) of GP-CBT-P against waitlist control. In addition to the three main assessment points, weekly measures of child and parental perfectionism over the intervention period were administered to the GP-CBT-P group. The study was prospectively registered with the Australian New Zealand Clinical Trials Registry (ANZCTR): ACTRN12623000396628. This trial protocol was approved by the Flinders University Human Research Ethics Committee (Project ID: 5816).

### Participants

Families with a child aged 7 to 12 years were eligible to participate if the parent identified the child as exhibiting elevated perfectionism that was negatively impacting psychosocial functioning. The initial screening involved questions such as, “Does your child set extremely high standards for themselves?” and “Do they pursue these standards even if it causes stress or self-criticism?” Families were recruited through schools, psychology clinics, paediatric services, libraries, child sport venues and social media platforms. Advertisements with QR codes were distributed via newsletters, emails, and social media posts. Interested families underwent eligibility screening by telephone or email. Exclusion criteria included organic brain damage, a history of complex trauma or recurrent self-injury or current suicidality. Randomization occurred after completion of the baseline assessment using computer-generated, quasi-random numbers.

### Power

We aimed to recruit 66 families (33 in each group) as this would provide 80% power to detect an effect size of 0.60 [26], which is consistent with findings from previous guided parent-delivered CBT interventions for child anxiety [24].

### Intervention

GP-CBT-P is a structured six-module program, described in Table 1, with two additional sections introducing the program and suggesting next steps following completion. Given the robust evaluations of the CBT-P protocol outlined in *Overcoming Perfectionism: A Self-Help Guide Using Cognitive Behavioural Techniques* [11] showing improvements across multiple variables and studies [13–15], we adapted it for parent delivery to ensure age-appropriate material and practical applicability. This was informed by consultation of three mothers (each reimbursed AUD$100) of a child with perfectionism, and previous co-design [24]. Based on feedback, several changes were made, including adding tips for involving children in the program and an overview to the introductory section, producing a handout to share with the child’s school about the program, creating simplified versions of homework, adding further psychoeducation on thoughts, feelings, and behaviours, and a video for children on the importance of mistakes.

**Table 1:** Content of the intervention modules

Session #	Content	Homework
Introduction	Who the program is for, what it involves, common concerns, and how to deliver it	Nil.
1	Psychoeducation: definition of perfectionism, the difference between high standards and perfectionism, myths of the advantages of perfectionism, exploring reasons to change.	Handout for child about perfectionism and its negative effects. Brief quiz for parent and child to complete together. Worksheet for the child to practise differentiating perfectionism from striving for high standards.
2	The connection between thoughts, feelings, and behaviours, what keeps perfectionism going, examining typical unhelpful thinking patterns and behaviours that maintain perfectionism and language parents can use to support their child to change their perfectionism.	Handout for child about the link between thoughts, feelings, and emotions. Worksheet for the child to practise applying this link. Parent practise praising their child for non-achievement related qualities while playing a game.
3	Parents practise setting up a behavioural experiment for their child’s perfectionism, and wellbeing tips (sleep, incorporating pleasant activities, and social media).	Handout and worksheet for child about behavioural experiments. Parent to support their child to set up and undertake a behavioural experiment. Handouts for child about sleep and social media.
4	Psychoeducation about self-criticism, identifying the critical voice, psychoeducation about self-compassion, ways to be more self-compassionate, and coming up with opportunities to model self-compassion for their child.	Handout for child on self-criticism and self-compassion. Self-compassion quiz for parent and child to complete together. Worksheet for child to practise self-compassion.
5	Behaviours that can reinforce perfectionism, such as procrastination, avoidance and checking, and using problem solving to address these maintaining behaviours.	Handout for child about procrastination. Psychoeducation handout and worksheet for child about problem-solving. Parent to look for opportunities to do problem-solving with their child.
6	Identifying various factors that impact self-worth to develop a self-worth pie chart, considering values and making values-based decisions, reflecting on the program, and coming up with action steps for after the program to continue assisting their child.	Handout for child about self-worth. Worksheet for parent and child to make the child’s self-worth pie chart and develop a plan for broadening this. Worksheet for parent/child to complete to review progress over the program.
Next steps	Suggestions for ongoing use of program content	Nil.

The program provides parents with the tools to reflect on and address perfectionistic tendencies, both in themselves and within their children. Parents were sent a PDF that included the module content and associated homework exercises one week in advance. Additionally, parents and children in the intervention group completed surveys immediately prior to each session, measuring weekly changes in perfectionism. Sessions were delivered online through 30-minute video calls between parents and an Honours level Psychology graduate trained in the intervention. To ensure fidelity to the program, the guide followed a standardized manual. Their role was to facilitate understanding, encourage problem-solving, and celebrate progress while refraining from offering direct advice. Children were not present during these sessions.

The intervention group was instructed to complete the six modules over a maximum of 12 weeks. The initial target timeline was six weeks but this was too condensed for some families to complete the homework exercises, leading to the introduction of a more flexible timeline. Despite this adjustment, the structured nature of the program was maintained, with all participants completing their T2 assessments approximately nine weeks post-randomization.

Each session began with collaborative agenda-setting. This included reviewing the parent’s experiences with homework, addressing current concerns, and focusing on the specific module for that session. Homework assignments were designed to help parents practice the skills in modules and to support their children in implementing strategies. Parents were encouraged to approach working through the homework with their child in manageable blocks over the week rather than adhering to a rigid schedule.

Families in the waitlist control group received the GP-CBT-P materials after completing both the T2 and T3 assessments. Retention strategies for both groups included follow-up email reminders for participants who did not complete assessments. For those showing passive withdrawal, follow-up emails continued for four weeks, with weekly reminders. In addition, families were reimbursed with an AUD$100 “Gift Pay” voucher upon completing the 18-week post-randomization assessment.

### Primary outcomes

The primary outcomes included feasibility metrics (e.g., number of families recruited and randomized), and acceptability (the number of parents completing modules and perceived credibility). Thresholds were chosen using benchmarking from brief guided parent-delivered cognitive behavioural therapy to children with anxiety disorders [23], where 136 children were randomised over a 2-year period, 19% did not complete the treatment. The 4-item Credibility and Expectations Questionnaire [CEQ; 27] was administered at mid- and post-intervention and benchmarked against ratings received for our online CBT-P with young adults [28]. Additionally, qualitative feedback was invited to gain further insights into experiences with the intervention.

### Secondary outcomes

#### Perfectionism in children and perfectionism in parents

For children we used a shortened version of the Almost Perfect Scale-Revised [29], which contains two subscales: High Standards (6 items) and Discrepancy (7 items). Items are rated on a 7-point scale ranging from 1 (*strongly disagree*) to 7 (*strongly agree*) with no set time frame. High Standards assesses adaptive striving while Discrepancy assesses the perception that high standards are not being met. Good internal consistency has been found for the subscales which are distinct from each other across ages 9 to 16 years [30]. For parents we used the 10-item Clinical Perfectionism Questionnaire (omitting the two reverse scored items which decrease internal reliability [31]), a self-report measure rated over the previous month. Internal reliability for all secondary outcomes is provided in Table 2.

**Table 2:** Change over time (ITT) - unshaded rows indicate intervention (*n* = 25) and shaded indicates control (*n* = 24)

Variable (internal reliability, baseline omega)	Baseline T1	End-Tx T2	6-week FU T3	Hedge’s g between group ES (95% CI) T3
High Standards (0.77)	5.43 (0.22)	5.28 (0.26)	5.48 (0.24)	0.14 (-0.41 to 0.69)
	5.42 (0.22)	5.20 (0.26)	5.31 (0.24)	
Discrepancy (0.82)	4.11 (0.81)	3.31 (0.84)^a^	3.23 (0.85)	0.19 (-0.37 to 0.74)
	4.38 (0.81)	4.34 (0.83)^b^	4.02 (0.84)	
Parental Clinical Perfectionism^1,2,3^ (0.76)	2.07 (0.09)	1.66 (0.13)^a^	1.75 (0.12)^a^	**0.65 (0.08 to 1.21)**
	2.19 (0.10)	2.26 (0.12)^b^	2.14 (0.12)^b^	
School Satisfaction^3^ (0.87)	6.76 (0.39)	7.58 (0.47)	7.61 (0.47)^a^	**0.66 (0.09 to 1.23)**
	7.10 (0.39)	6.31 (0.43)	6.08 (0.45)^b^	
Happiness (1 item only)	6.48 (1.90)	6.74 (1.93)	7.54 (1.91)^a^	0.16 (-0.39 to 0.71)
	6.87 (1.90)	6.06 (1.93)	6.01 (1.91)^b^	
Self-efficacy Academic^3^ (0.70)	3.59 (0.12)	3.71 (0.14)	3.84 (0.17)^a^	**0.67 (0.10 to 1.24)**
	3.65 (0.12)	3.42 (0.14)	3.25 (0.18)^b^	
Perceived Stress^3^ (0.83)	1.22 (0.34)	1.02 (0.35)	0.94 (0.35)^#^	0.16 (-0.39 to 0.71)
	1.09 (0.35)	1.17 (0.35)	1.22 (0.35)^#^	
Depression^3^ (0.90)	1.12 (0.12)	0.94 (0.16)	0.67 (0.14)^a^	**0.82 (0.25 to 1.40)**
	1.06 (0.12)	1.23 (0.16)	1.25 (0.14)^b^	
Weight and Shape Concern (0.93)	1.80 (0.18)	1.58 (0.19)^a^	1.52 (0.24)	**0.58 (0.02 to 1.15)**
	1.95 (0.19)	2.18 (0.18)^b^	2.21 (0.23)	

Note: Superscripts indicate significant: ^1^ main effect of time; ^2^ main effect of treatment; ^3^ time x treatment interaction; Superscripts ^a b^ indicate least significant difference (equivalent to no adjustments) between the two groups at this time point; ^#^ *p*=.055; T1, T2, T3 refer to Time 1, Time 2, Time 3; bolded effect size (ES) and confidence intervals (CI) indicate that the CI do not cross zero

#### Mental health in children

The 13-item Perceived Stress Scale: Children [32] is measured on a 4-point scale (Never to a Lot) over the previous week, with an example item including: “How often do you feel worried about your grades or school?” The scale effectively discriminates between children aged 5 to 18 years with and without known anxiety disorders/stress [32].

#### Centre for epidemiological studies depression scale for children

This 20-item self-report depression inventory [33] is commonly used to measure depression in children aged 6 to 17 years over the previous week. Each response to an item is scored as follows: 0 = “Not at All” 1 = “A Little” 2 = “Some” 3 = “A Lot”.

#### Eating disorder-15, parents/caregivers report

We used the 10 items that relate to weight and shape concern over the previous week (e.g., My child … compares their body negatively to others, feels nervous or uptight about their weight), omitting the behavioural items given children were not identified as having an eating disorder. Excellent reliability, discriminant and concurrent validity, and good sensitivity to change early in treatment have been obtained for these 10 items in children aged 8 to 18 years [34].

#### Approach to valued goals in children

We used three indicators of this construct. The first is a measure of school satisfaction and happiness tested in children aged 10 years [35]. Questions included: Satisfaction with school, comprising six indicators, introduced by a question regarding the level of satisfaction: e.g., “How satisfied are you with each of the following things in your life? The response options were coded from 0 “Not at all satisfied” to 10 “Totally satisfied”. Second, happiness was assessed by asking: “Overall, how happy have you been feeling during the last two weeks?”, with response options from 0 “Not at all happy” to 10 “Totally happy”. Third, we used the 8-item academic self-efficacy subscale (e.g., “How well do you succeed in finishing all your homework every day?”) from the Self-efficacy Questionnaire for Children [36], validated for ages 12 to 19 years. Each 5-point item was measured with responses ranging from “Not at all” to “Very well”. There was no time frame set for any of the items.

### Statistical analysis plan

Linear mixed models were used at three assessment points: baseline, 9 weeks, and 18 weeks post-randomization. The primary focus was to estimate between-group effect sizes, along with their 95% confidence intervals. The Reliable Change Index was also used to assess meaningful individual changes in outcome measures, including any potential deterioration.

## Results

### Feasibility

**Fig. 1:**
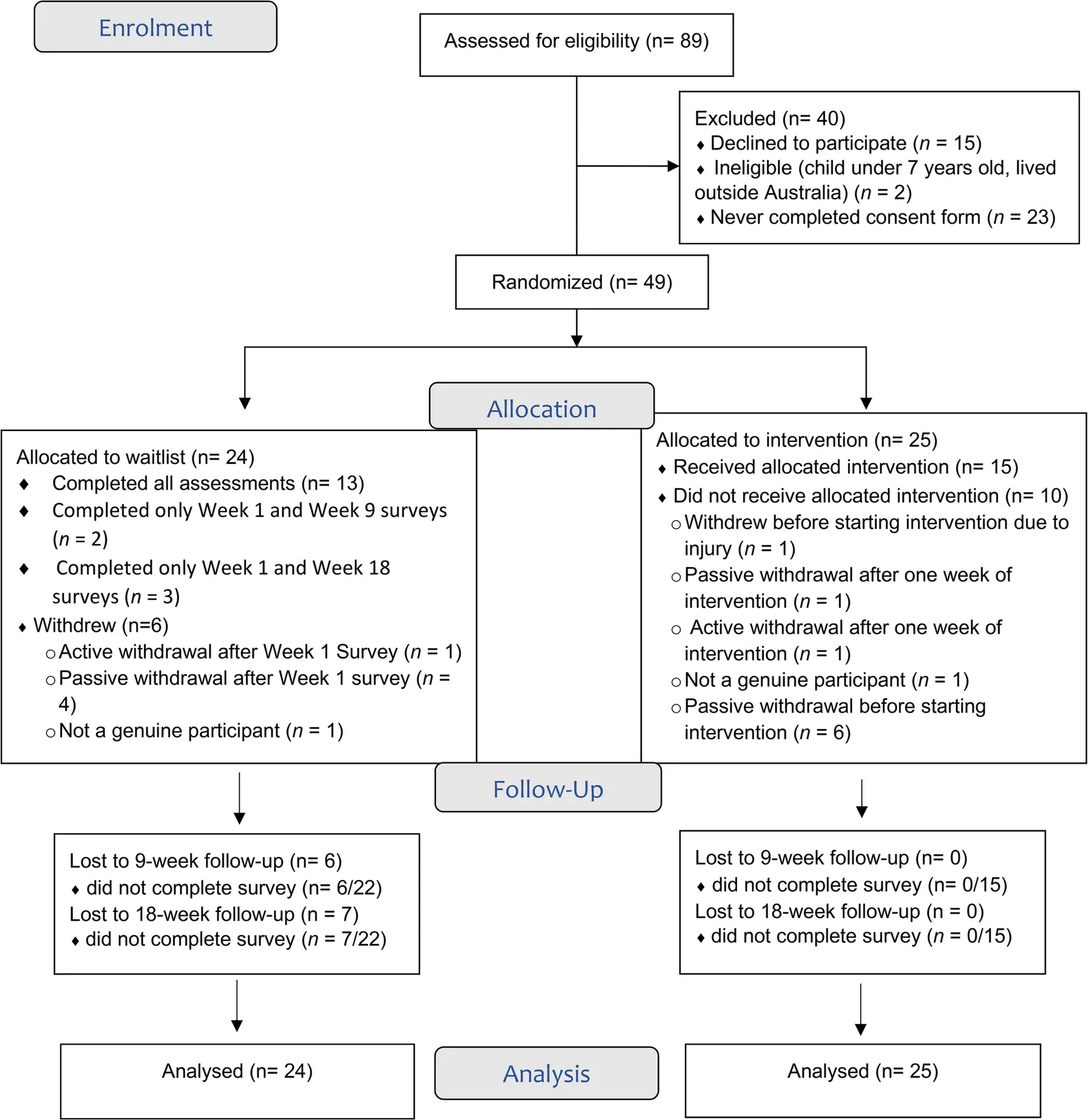
CONSORT flow diagram

As shown in Fig. 1, over 14 months, a total of 89 families expressed interest, 87 were deemed eligible, 49 (55%) converted to randomisation, with 25 in GP-CBT-P and 24 in the waitlist control. The mean age of the child was 9.90 years (SD = 1.37) ranging from 7 to 12. The average monthly rate of recruitment was 3.5 compared to 5.7 reported previously for anxious children [23].

### Acceptability

Fourteen families discontinued participation; nine (36%) from the GP-CBT-P group (twice as high as previously reported for anxious children [23]) and five (21%) from the waitlist control group. 60% of parents in the intervention group completed all six sessions. The qualitative feedback, summarised in Table 3, was generally positive. Parents particularly noted that the pace of one module a week, as initially commenced, was too much due to other commitments, but also felt that it was optimally situated in the school term when problems with perfectionism were most notable. Children were less enthusiastic about having to complete the questionnaires.

**Table 3:** Qualitative feedback from parents and children at end of treatment

	What aspects of the program did you like the most?	What aspects of the program did you like the least?
Parent	The tools given through each module to be able to help my child and guidance on what to do.	Doing the program over the holiday period it was hard to assess the effectiveness when a lot of my child’s relevance was more in relation to school.
	*Identifying the specifics around perfectionism…while the concepts around reducing these areas for greater satisfaction in life seem simple - when it comes to your child it is hard to articulate so that has been helpful*	*Some of the concepts are bit hard to understand for a 7-year-old or procrastination. The questionnaire is a bit wordy to explain to a 7-year-old which may affect its accuracy.*
	The educational materials for parents. My son actually read them himself.	Too fast paced; with our busy schedule, the timeline was rushed.
	*That it catered to younger and older kids with the scenarios. Simple worksheets with activities to assist kids. The information given at the beginning of each unit was informative to provide insight into which areas of perfectionism can be… identified in my child.*	*There were some interesting questions about body image and food that I wished went deeper than just looking at food in a negative light. Food and eating for my daughter is a big deal… she has a negative body image despite being very healthy.*
	The homework modules, very well written and appropriate for 7-year-old. Materials and information that was provided and practical tips.	Fear I was doing the program incorrectly or not effectively enough, difficulty figuring out how to tailor to my child for best results.
Child	Doing activities with my mum.	It’s a bit hard.
	*Finding out what the word procrastinate means and seeing some ideas on how to not do it*	*The first module was hard for me.*
	All of it; Learning new things; Ideas to help my mindset; Module 5; The self-worth pie.	Self-compassion and talking to myself as a friend.
	*Some of the scenarios; the problems that Adam and Lucy had (case studies) and checklists*	*Having to do more homework.; it’s like schoolwork.*
	I liked answering questions about being stressed and overcompensating. It made me think about how I respond in stressful situations.	Answering the questions at the end each week; answering all the questions; sometimes I didn’t like some questions about eating habits and sleeping.

Ratings on the CEQ were examined in the intervention group (*n* = 15). There were no significant changes in ratings between the two time periods. At week three parents reported a mean (SD) of 7.33 (1.11) out of 9 for logicality, 5.67 (1.54) out of 9 for capacity for potential success, 6.80 (1.86) out of 9 for confidence to recommend to a friend, and 42 (23.66) out of 100 for expected improvement in functioning. These were, on average, slightly higher than received previously with young adults [28], respectively 6.17, 5.67, 5.48, 45.23.

### Secondary outcomes

We compared participants with missing data (*n* = 22) to those with complete data (*n* = 26) across all three waves to examine change over time. Only one baseline variable was significantly different between the two groups, parent perfectionism, where those with higher levels of perfectionism (*M* = 2.30, *SD* = 0.37) were more likely to have incomplete data than those with lower perfectionism (*M* = 1.99, *SD* = 0.51). Thus, we cannot conclude that data are missing at random.

First, we examined change over time between the groups, reported in Table 2. There were no differences between groups at baseline. The intent-to-treat analysis showed parents in GP-CBT-P had significantly lower perfectionism at T2 and T3 than control with large and moderate between-group effect sizes. No significant between-group differences were found for child perfectionism or happiness. Compared to control, children receiving GP-CBT-P reported significantly higher school satisfaction and academic self-efficacy, and lower stress, depression and weight/shape concern; all T3 effect sizes were moderate.

**Fig. 2:**
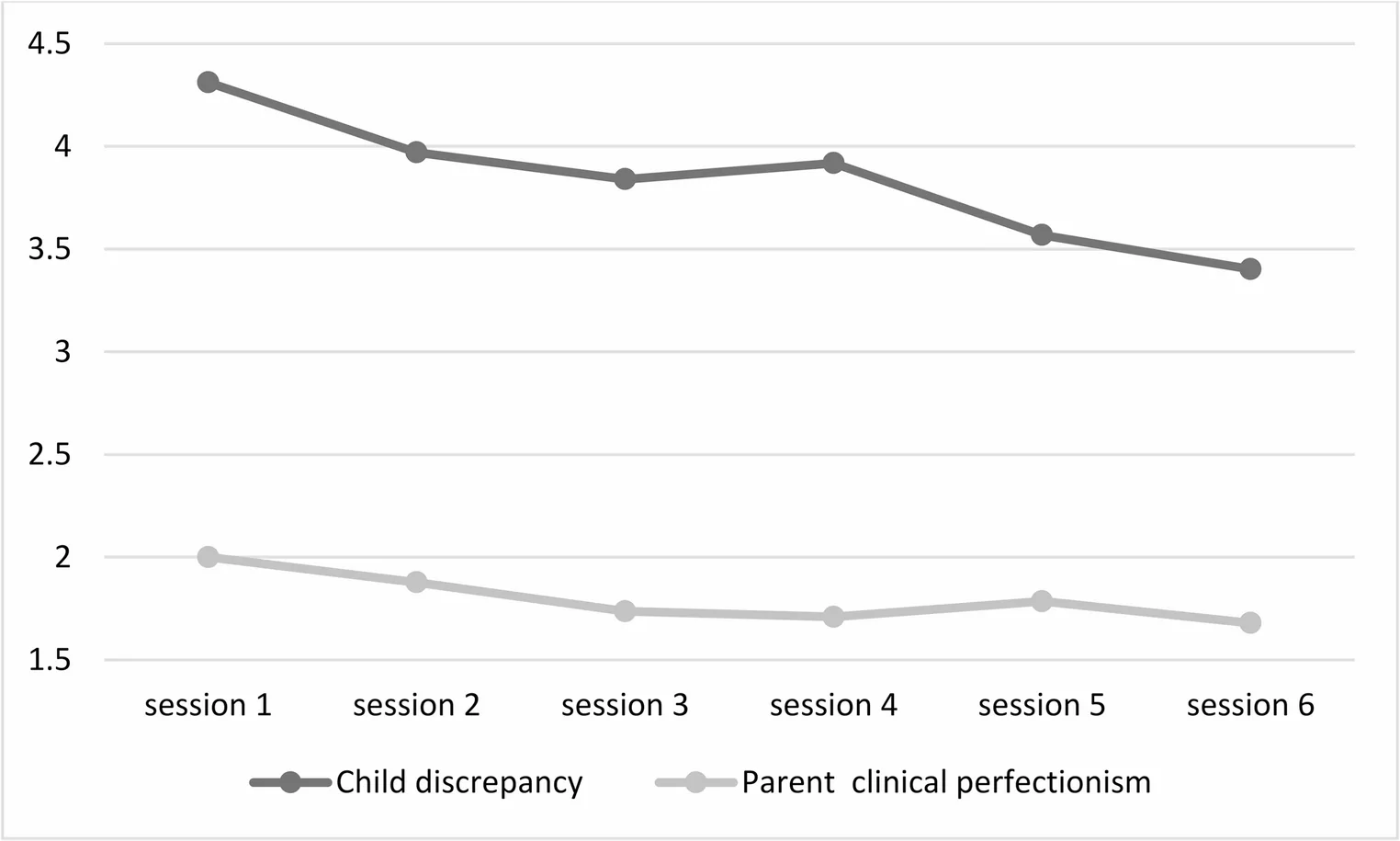
Change in sessional perfectionism in children and parents in the intervention group

Second, we examined sessional changes over time in perfectionism in children and parents. Our measure of adaptive striving in children did not decrease significantly over time *F*(5, 14.51) = 2.77, *p*=.06, while self-reported discrepancy did, *F*(5, 14.55) = 7.59, *p*=.001 (corrected Hedge’s *g* = 0.56, 95% CI: 0.16–0.96). Parent-reported perfectionism also decreased significantly over time, *F*(5, 13.77) = 6.53, *p*=.003 (*g* = 0.66, 95% CI: 0.19–1.13). The rate of these declines is illustrated in Fig. 2.

Reliable change is summarized in Table 4, with the most striking feature being that no participants receiving GP-CBT-P experienced deterioration whereas between 8% and 23% of the participants in the control group experienced deterioration for 8 out of 9 variables. Reliable improvement was experienced in both groups, with the control group showing improvement on perfectionism variables only, and the GP-CBT-P participants showing the most notable improvement for happiness.

**Table 4:** Reliable change between T1 and T3

Variable	RCI	*N* improved Intervention *n* = 15	*N* deteriorated Intervention *n* = 15	*N* improved Control *n* = 13	*N* deteriorated Control *n* = 13
High standards	1.76	0	0	1 (8%)	0
Discrepancy	2.82	0	0	2 (16%)	1 (8%)
Parent perfectionism	0.67	1 (7%)	0	3 (23%)	1 (8%)
School satisfaction	3.58	1 (7%)	0	0	2 (16%)
Happiness	3.17	3 (20%)	0	0	2 (15%)
Academic self-efficacy	1.30	0	0	0	3 (23%)
Stress	0.91	1 (7%)	0	0	2 (16%)
Depression	1.21	0	0	0	2 (16%)
Weight/shape concern	1.27	0	0	0	2 (16%)

## Discussion

Although this intervention decreased parental rather than child perfectionism, it improved children’s problems commonly associated with perfectionism, including mental health problems and reduced approach to valued goals. While credibility and expectations of the intervention were acceptable, a lower rate of recruitment and higher dropout compared to a study of childhood anxiety [23] was indicated. The main obstacles to feasibility and acceptability included parental perfectionism and the time required to commit to the intervention in a busy family setting. It may be that some introductory material is required to explain the wisdom of investing time now in helping a child better learn to manage perfectionism to prevent longer-term harmful impacts on mental health and academic endeavours. The use of enhanced introductory material that incorporates the current results and explains how such an intervention can help the child’s broader functioning may also be helpful to motivate parents to prioritise use of the material amongst all other competing demands in the family.

Despite being slightly underpowered, we found moderate effect size differences favouring the intervention over waitlist control for parental perfectionism and for school satisfaction, academic self-efficacy, depression and weight/shape concern in children. These outcomes, along with the reliable deterioration noted across variables in the control but not the intervention group, illustrate the broad negative impacts of perfectionism on children, consistent with the literature [7–9]. In our intervention group we did not see a decrease of High Standards [26] in children, which assesses adaptive striving, but as intended, there were significant moderate decreases in our weekly measures of Discrepancy [26], considered a harmful aspect of perfectionism. This equated to a significant difference favouring the intervention group at end of treatment, however at follow-up this significant difference was not maintained, with a 0.88 and 0.36 decrease in perfectionism in the intervention and control groups respectively. This reflects a need for a larger, fully powered study to investigate the longer-term effects of the intervention on perfectionism.

In the absence of a decrease in perfectionism in children, coupled with the early and significant decline in parental perfectionism that preceded improvements for children, our results may indicate that changes in parental perfectionism are a key driver of beneficial change in children. Given evidence that parental anxiety is associated with poorer treatment outcomes for childhood anxiety disorders [37], developing a modular intervention that can target both parental (if present) and child perfectionism could offer substantial benefits. It should be noted, however, that it is hard to disentangle the effect of targeting perfectionism from the effect of having the parents more involved in their child’s wellbeing over the period of the intervention.

The results should be interpreted in the context of four key limitations. First, our data cannot be interpreted as missing at random as parents with higher perfectionism were more likely to have incomplete data than those with lower perfectionism. Perfectionism is highly correlated with procrastination and avoidance [11], which may have impacted completion of assessments, or completion of the modules. For this reason, we only report an intent-to-treat approach to data analysis. Second, not attaining the full sample size indicated by our power analysis coupled with around with one-third of our sample ceasing treatment prematurely, means our intent-to-treat analysis should be interpreted cautiously. Third, the outcomes were limited to self-report, and it would be of interest in future research to examine teacher reports. Fourth, this intervention may rely heavily on parental ability to fully engage in the material, which may be reduced by their perfectionistic tendencies, which could limit the effectiveness or fidelity of implementation.

In summary, parent delivery of CBT for perfectionism to their children may be a useful intervention for parental perfectionism and children’s emotional wellbeing which appears to be stronger than universal classroom-delivered lessons [16, 17]. Previous evaluations have not examined academic indicators that we expect to be impaired by perfectionism [8], so our findings showing improvement for these indicators are important to replicate. It may be that changes to the parent’s way of relating to their children offers a more powerful mechanism for improving child mental health, consistent with cross-sectional evidence [e.g., 21]. Further research should examine whether targeting parental perfectionism only is sufficient to create meaningful or lasting change in children’s perfectionism. It is also worth exploring whether this model may be more suitable for prevention rather than treatment, or for children with less severe perfectionism.

## Data Availability

The data that support the findings of this study are available from the corresponding author upon reasonable request.

## Declarations

### Preregistration

The study was prospectively registered on 19/4/23 with the Australian New Zealand Clinical Trials Registry (ANZCTR): ACTRN12623000396628.
